# Huntingtin-associated protein 1-associated intracellular trafficking in neurodegenerative diseases

**DOI:** 10.3389/fnagi.2023.1100395

**Published:** 2023-02-07

**Authors:** Xingxing Chen, Enhao He, Chonglin Su, Yan Zeng, Jiang Xu

**Affiliations:** ^1^Brain Science and Advanced Technology Institute, Hubei Province Key Laboratory of Occupational Hazard Identification and Control, School of Medicine, Wuhan University of Science and Technology, Wuhan, Hubei, China; ^2^Geriatric Hospital Affiliated to Wuhan University of Science and Technology, Wuhan, Hubei, China; ^3^Hubei Key Laboratory of Nerve Injury and Functional Reconstruction, Tongji Hospital, Tongji Medical College, Huazhong University of Science and Technology, Wuhan, Hubei, China

**Keywords:** Huntingtin-associated protein 1, neurodegenerative diseases, intracellular trafficking, aging, selective neurodegeneration

## Abstract

Huntingtin-associated protein 1 (HAP1), the first identified HTT-binding partner, is highly expressed in the central nervous system, and has been found to associated with neurological diseases. Mounting evidence suggests that HAP1 functions as a component of cargo-motor molecules to bind various proteins and participates in intracellular trafficking. It is known that the failure of intracellular transport is a key contributor to the progression of neurodegenerative disorders (NDs) including Alzheimer’s disease (AD), Huntington’s disease (HD), Parkinson’s disease (PD), amyotrophic lateral sclerosis (ALS), spinal and bulbar muscular atrophy (SBMA) and spinocerebellar ataxia (SCA). The link between HAP1 and various NDs is supported by growing evidence. This review aims to provide a comprehensive overview of the intracellular trafficking function of HAP1 and its involvement in NDs.

## Introduction

Neurodegenerative diseases (NDs) including Alzheimer’s disease (AD), Huntington’s chorea (HD), Parkinson’s disease (PD), amyotrophic lateral sclerosis (ALS), spinal and bulbar muscular atrophy (SBMA) and different types of spinocerebellar ataxia (SCA) share important pathological features characterized by the age-dependent and slowly progressive degeneration of selectively vulnerable nerve cells. NDs are caused by the accumulation of misfolded proteins, though they exhibit heterogeneous clinical and pathological phenotypes (Dugger and Dickson, 2017). While the pathogenic mechanisms of NDs are poorly understood, many lines of evidence point to a role of disrupted neuronal trafficking during the development of these diseases (Suzuki et al., 2006; Schreij et al., 2016; Burk and Pasterkamp, 2019; Singh and Muqit, 2020; Blackstone et al., 2021; Liu et al., 2021). The defects in intracellular trafficking typically result in mislocalization of proteins and the accumulation of undegraded proteins in neurons.

Huntingtin-associated protein 1 (HAP1) was first identified in yeast two-hybrid screens to interact with huntingtin (HTT), a protein known to cause HD when there is increased polyglutamine (polyQ) expansion in HTT (Li et al., 1995). Unlike HTT that is ubiquitously expressed in the brain and peripheral tissues, HAP1 is mainly enriched in neurons (Li et al., 1995; Gutekunst et al., 1998), suggesting that HAP1 may be associated with selective neurodegeneration in HD. In the rodent animals, HAP1 is more abundant in the limbic and hypothalamic regions (Fujinaga et al., 2004). However, our recent study indicates that HAP1 is ubiquitously expressed in different regions in the monkey and human brains, such as cortex, striatum (putamen and caudate), hippocampus and hypothalamus, indicating that HAP1 is expressed in a species-dependent manner (Chen et al., 2022). It has been reported that HAP1-immunoreactive products are exhibited as cytoplasmic puncta or “stigmoid bodies” (STBs), a special cellular structure ranged from 0.5 to 3 μm in diameter (Gutekunst et al., 1998; Li et al., 1998a). Although HAP1 immuno-positive puncta are also seen in the monkey brain, most of the puncta were often small (< 1 μm in diameter) (Chen et al., 2022). STBs appear to be formed by intracellular microtubule-dependent multiple fusion of small HAP1 inclusions (Fujinaga et al., 2009). HAP1 in the rat consists of two isoforms (HAP1A and HAP1B) that differ in their C-terminal sequence (Li et al., 1995). Human HAP1 has a major form (molecular weight is about 75kD) that is very similar to rat Hap1A (Li et al., 1998b). Increasing evidence suggests that HAP1 is a component of cargo-motor molecules that binds various proteins and participates in intracellular trafficking (Table 1). Abnormal interaction of mutant proteins with HAP1 affects the intracellular transport of selected molecules, which can critically contribute to the pathogenesis of neurodegeneration.

**Table 1:** HAP1-interacting proteins.

Name	Function	Refs
Huntingtin (HTT)	Scaffold protein	Li et al. (1995)
The p150Glued subunit of dynactin (p150Glued)	Microtubule-dependent transporter	Engelender et al. (1997)
Kinesin light chain 2 (KLC2)	Molecular motor	McGuire et al. (2006)
Kinesin family motor protein 5 (KIF5)	Molecular motor	Twelvetrees et al. (2010)
Rho-GEF/Kalirin-7 (Duo)	GDP-GTP exchange factor	Colomer et al. (1997)
Brain-derived neurotrophic factor (BDNF) and precursor of BDNF (proBDNF)	Neurotrophin factor	Gauthier et al. (2004) and Wu et al. (2010)
Neuron-specific phosphoprotein (synapsin I)	Vesicular trafficking	Mackenzie et al. (2016)
Hepatocyte growth factor-regulated tyrosine kinase substrate (Hrs)	Endosometo-lysosome trafficking	Li et al. (2002)
Epidermal growth factor receptor (EGFR)	Membrane receptor	Li et al. (2002)
γ-aminobutyric acid type A receptor (GABAAR)	Membrane receptor	Kittler et al. (2004)
Tropomyosin-related kinase A receptor tyrosine kinase (Trk A)	Membrane receptor	Rong et al. (2006)
type 1 inositol (1,4,5)-trisphosphate receptor (InsP3R1)	Membrane receptor	Tang et al. (2003)
Transcriptional repressor RE1-silencing transcription factor/ neuron-restrictive silencer factor (REST/NRSF)	Transcription factor	Shimojo (2008)
The basic helix–loop–helix transcription factor (NeuroD)	Transcription factor	Marcora et al. (2003)
Androgen receptor (AR)	Nuclear receptor	Takeshita et al., 2006
Glucocorticoid receptor (GR)	Nuclear receptor	Chen et al. (2020)
Abelson-helper integration site 1(AHI1)	Scaffold protein	Sheng et al. (2008)
Amyloid precursor protein (APP)	Transmembrane precursor protein	Yang et al. (2012)
TATA binding protein (TBP)	Transcription factor	Prigge and Schmidt (2007)
Ataxin-3	Deubiquitination enzyme	Takeshita et al. (2011)

### Huntingtin-associated protein 1-associated intracellular trafficking

As a cytosolic protein in neurons, HAP1 lacks conserved transmembrane domains and nuclear localization signals. It contains a coiled-coil domain in the middle region and multiple N-myristoylation sites which are found in a variety of proteins associated with membrane proteins and involved in intracellular trafficking (Li and Li, 2005), Consistently, the electron microscopy results show that HAP1 is distributed in microtubules and membranous organelles (Gutekunst et al., 1998). Intracellular anterograde and retrograde transport of membrane organelles requires the molecular motors kinesin and dynein. The motor protein dynein, which is involved in retrograde transport, is required to drive vesicles along microtubules with the assistance of microtubule transporter dynactin. HAP1 might be involved in intracellular trafficking *via* its interaction with p150Glued that is the largest subunit of dynactin and mediates the binding between microtubules and dynein (Engelender et al., 1997; Tempes et al., 2020). HAP1 interacts with p150Glued to form a stable complex that may mediate the microtubule-dependent retrograde of membranous organelles (Engelender et al., 1997). HAP1 also participates in anterograde transport in association with kinesin, which consists of two heavy chains (KHC) and two light chains (KLC) for moving along microtubules with the energy generated by ATP hydrolysis. There is an interaction between HAP1 and kinesin light chain 2 (KLC2), and deletion of HAP1 results in significant suppression of kinesin-dependent amyloid precursor protein (APP) transport in neurons (McGuire et al., 2006). Further, the association of HAP1 with p150Glued and KLC can be reduced by the phosphorylation of the C-terminus of HAP1A, and increased when HAP1A is dephosphorylated (Rong et al., 2006). In addition, HAP1 associates with Rac1 guanine nucleotide exchange factor Kalirin-7/Duo that has characteristics of membrane cytoskeletal proteins (Colomer et al., 1997). The interactions of HAP1 with these cytoskeletal proteins allow HAP1 to act as a scaffold protein to participate in intracellular trafficking, which is supported by increasing evidence.

First, HAP1 is reportedly associated with synaptic vesicles (Gutekunst et al., 1998). Studies have shown an important role of HAP1 in the vesicular transport of brain-derived neurotrophic factor (BDNF) and proBDNF, a precursor of BDNF (Gauthier et al., 2004; Wu et al., 2010; Yang et al., 2011). BDNF is a member of the neurotrophin family regulating neuronal development and plasticity. It is synthesized as proBDNF that is cleaved in the Golgi or immature secretory vesicles to form mature BDNF. BDNF is transported anterogradely in neurons and released from the nerve terminal in an activity-dependent manner. HTT, HAP1, and p150Glued form a complex that is involved in vesicular transport of BDNF along microtubules (Gauthier et al., 2004). BDNF transport is blocked when HAP1 level is reduced by small-interfering RNA (siRNA). HAP1 also participates in axonal transport and activity-dependent release of proBDNF by interacting with the BDNF prodomain (Wu et al., 2010). HAP1 and proBDNF form a complex with sortilin to be engaged in Golgi-endosome transport, preventing proBDNF degradation and modulating its targeting to endosomes, microtubules, and neuritis (Yang et al., 2011). The regulation of microtubule-dependent vesicles transport by HAP1 may involve synapsin I, a neuron-specific phosphoprotein that is associated with synaptic vesicles during neurotransmitter release to regulate synaptic plasticity and neuronal development (Cesca et al., 2010; Mackenzie et al., 2016). These findings suggest a direction for investigation of how HAP1 regulates synaptic function.

Second, HAP1 appears to be important for the stability and recycling of membrane receptors. HAP1 was reported to interact with hepatocyte growth factor-regulated tyrosine kinase substrate (Hrs), a key protein for endosometo-lysosome trafficking of membrane receptors (Li et al., 2002). Moreover, HAP1 is able to stabilize the internalized membrane receptors, such as epidermal growth factor receptor (EGFR), γ-aminobutyric acid type A receptor (GABAAR, the major inhibitory receptor in the brain) and tropomyosin-related kinase A receptor tyrosine kinase (TrkA), a nerve growth factor (NGF) receptor required for neurite outgrowth (Li et al., 2002; Kittler et al., 2004; Rong et al., 2006). When the interaction of HAP1 with microtubule-dependent transporters is suppressed, these membrane receptors cannot be efficiently transported to the appropriate cellular sites and are instead targeted to the lysosomes for degradation, resulting in a decrease of their levels and defective neural activity. HAP1 also binds to other membrane receptors, such as the type 1 inositol (1, 4, 5)-trisphosphate receptor (InsP3R1), an intracellular Ca^2+^ channel localized in the endoplasmic reticulum. The InsP3R1 carboxy terminus binds HAP1A and HTT to form the InsP3R1/HAP1A/HTT ternary complex (Tang et al., 2003). HAP1 can affect InsP3R1-mediated Ca^2+^ release by regulating the binding of InsP3R1 and HTT (Tang et al., 2004; Arteaga-Bracho et al., 2016). All these findings provide an insight into the regulation of neuronal function by HAP1 through its effect on membrane receptors.

Third, HAP1 is involved in the nuclear translocation of transcription factors. HAP1, HTT, p150Glued, the transcriptional repressor RE1-silencing transcription factor/neuron-restrictive silencer factor (REST/NRSF), and the REST/NRSF-interacting LIM domain protein (RILP) were found to form a complex involved in the translocation of REST/NRSF into the nucleus, a process in which HAP1 controls REST/NRSF cellular localization in neurons (Shimojo, 2008; Zhu et al., 2019). HAP1 acts as a scaffold protein to provide binding sites for two or more components of a signaling pathway so they can be tightly linked together (Smith and Scott, 2002; Marcora et al., 2003). For example, HTT interacts with NeuroD, a basic helix–loop–helix transcription factor important for neuronal development and survival *via* HAP1. HAP1 and HTT facilitate activation of NeuroD by mixed-lineage kinase 2 (MLK2) (Marcora et al., 2003). NeuroD activation is correlated with its translocation from the cytoplasm to the nucleus (Petersen et al., 2002). In addition, HAP1 appears to associate with nuclear translocation with steroid hormone receptors that are ligand-dependent transcription factors and include androgen receptor (AR) and glucocorticoid receptor (GR) (Fujinaga et al., 2011). In line with this, it has been shown that HAP1 interacts with AR (Takeshita et al., 2006). Our recent findings also suggest that HAP1 interacts with GR and can stabilize the level of GR, indicating that HAP1 may be involved in the regulation of stress in the central nervous system (Chen et al., 2020). Consistent with this finding, the Abelson-helper integration site 1(AHI1), which forms a stable protein complex with HAP1 (Sheng et al., 2008), also regulates the nuclear translocation of GR (Wang et al., 2021).

### The role of HAP1 in HD

**Figure 1:**
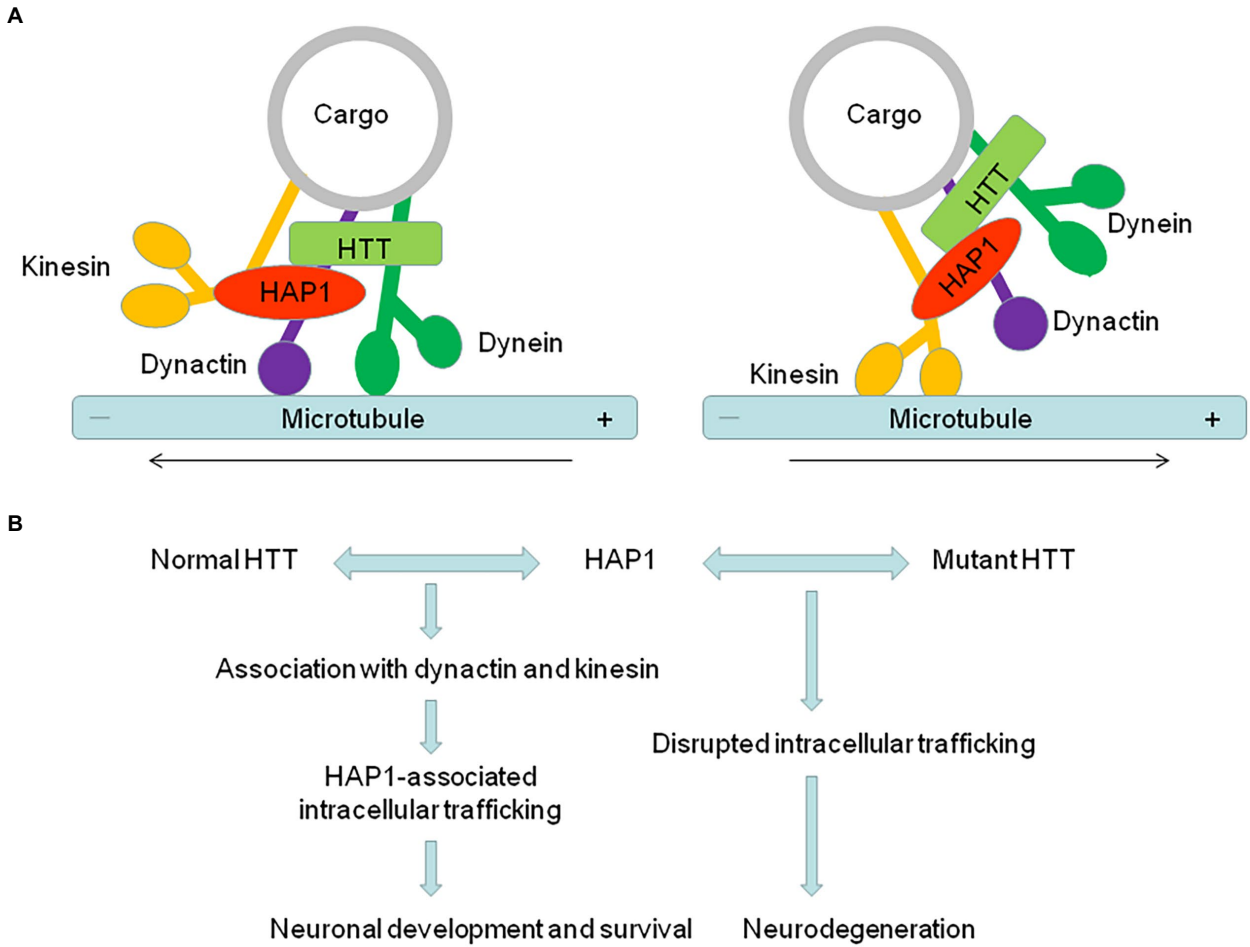
The abnormal interaction of HAP1 with mutant HTT is an important contributor to the pathogenesis of neurodegeneration in HD. **(A)** HAP1 is involved in intracellular trafficking *via* its interaction with dynactin and kinesin. The dynactin complex promotes retrograde trafficking, while kinesin promotes anterograde trafficking. **(B)** HAP1-dependent intracellular trafficking important for neuronal functions is affected by the abnormal interaction of HAP1 with mutant HTT, therefore resulting in neurodegeneration in HD.

HD is a monogenetic neurodegenerative disorder, with autosomal dominant inheritance, and displays unrelenting progressive motor, psychiatric and cognitive symptoms that are caused by severe neuronal loss in the striatum, hippocampus, and cerebral neocortex (Dom et al., 1976; Hedreen and Folstein, 1995; Murphy et al., 2000). The expansion of CAG trinucleotide repeat in the causative HTT gene on chromosome 4 leads to a prolonged polyQ repeat of variable length in the N-terminal region of HTT (Walker, 2007; McColgan and Tabrizi, 2018), which causes HTT to aggregate and accumulate in the nucleus and cytoplasm (DiFiglia et al., 1997; Gutekunst et al., 1999). Although HTT is expressed widely in the central nervous system (Li et al., 1993; Sharp et al., 1995; Bhide et al., 1996), mutant HTT (mHTT) with the expanded polyQ repeat causes selective neurodegeneration in the brain (Vonsattel et al., 1985; Wang et al., 2014; Arteaga-Bracho et al., 2016). HAP1 interacts with polyQ-expanded HTT more tightly in a polyQ length dependent manner (Li et al., 1995). In particular, our recent research shows that HAP1 is correlatively expressed with HTT in monkey and human brains (Chen et al., 2022), suggesting that abnormal protein–protein interactions may be an important contributor to the selective neuronal pathogenesis of HD (Figure 1).

HAP1 and HTT are associated with trafficking proteins to participate in intracellular transport. Since HAP1 binds mHTT more tightly than normal HTT, HAP1-dependent transport of various vesicles or receptors could be affected by the abnormal interaction of HAP1 with mHTT. For example, mHTT can dissociate the HTT/HAP1/p150Glued complex from microtubules, together with the weakened interaction between HAP1 and proBDNF in HD, affecting the transport and release of BDNF (Gauthier et al., 2004; Wu et al., 2010). Decreased BDNF transport results in the loss of neurotrophic support and increased neuronal toxicity. Consistent with that, mHTT reduces the association of HAP1 with p150Glued and KLC and thereby decreases the intracellular level of TrkA, whose internalization and trafficking are required for neurite outgrowth (Rong et al., 2006). In HD mouse model, the transport of GABAAR, which mediates inhibitory postsynaptic currents (IPSCs), and α-Amino-3-hydroxy-5-methyl-4-isoxazolepropionic acid (AMPA) receptor, which mediates excitatory postsynaptic currents (EPSCs), is also impaired. HAP1 interacts with the kinesin family motor protein 5 (KIF5), and this interaction-mediated anterograde transport of GABAARs and GluR2-containing AMPARs along microtubules in dendrites is disrupted by mHTT, leading to disrupted excitatory/inhibitory balance in HD (Twelvetrees et al., 2010; Mandal et al., 2011; Yuen et al., 2012). Moreover, mHTT alters the InsP3R1/HAP1A/HTT complex and subsequently sensitizes InsP3R1 activation to facilitate Ca^2+^ release in medium spiny striatal neurons (MSN) (Tang et al., 2003), providing a molecular mechanism to explain the alterations of intracellular Ca^2+^ signaling that are believed to play a significant role in the pathogenesis of HD (Kolobkova et al., 2017). HAP1 also seems to be involved in other neuropathological changes in HD. Disruption of autophagy has been implicated in HD, in which both soluble and aggregated polyQ-HTT are cleared by autophagy (Ravikumar et al., 2002; Qin et al., 2003). HAP1, as a dynein-activating adaptor, drives autophagosomal transport by binding the dynein–dynactin complex *via* canonical and noncanonical interactions (Cason and Carman, 2021). The regulation of autophagosome dynamics by HAP1 is disrupted by mHTT, leading to defective cargo degradation and the accumulation of polyQ-HTT observed in the neurons in HD (Wong and Holzbaur, 2014). These different lines of evidence indicate that mHTT affects various kinds of HAP1-dependent intracellular trafficking important for neuronal functions, therefore playing an important role in neurodegeneration.

HAP1 together with other HTT interacting proteins may be involved in the neuronal degeneration in the striatum, a region that is most affected in HD (Vonsattel et al., 1985; Vonsattel and DiFiglia, 1998). The small G protein Rhes, an E3 ligase for attachment of small ubiquitin-like modifier, is enriched in the striatum and binds selectively to mHTT and enhances sumoylation of mHTT (Subramaniam and Snyder, 2011). Sumoylation decreases the formation of mHTT aggregates and promotes cell death as sumoylated mHTT is more toxic (Subramaniam and Snyder, 2011; Soares et al., 2022). HAP1 deficiency in adult HD knock-in (KI) mouse brains caused more Rhes to bind mHTT and increased the level of sumoylated mHTT, leading to neuronal loss in the striatum (Liu et al., 2020). Neuronal loss induced by HAP1 deletion appears to require the presence of mHTT, consistent with our recent observation that deletion of HAP1 exacerbated neurotoxicity of mHTT in the organotypic brain slices of adult monkeys (Chen et al., 2022). However, how the interaction of mHTT and HAP1 mediates neuronal dysfunction in the primate brains needs to be further studied.

### The role of HAP1 in AD

Alzheimer’s disease is the most common form of neurodegenerative dementia worldwide, and its prevalence continues to grow with increase in the aged population worldwide. The initial stages of AD are characterized by deficits in the ability to encode and store new memories, followed by progressive cognitive and behavioral changes that occur at the later stages (Soria Lopez et al., 2019). The main pathological changes in AD include extracellular beta-amyloid (Aβ) plaques accumulation and intracellular neurofibrillary tangles, leading to synaptic damage or loss and neurodegeneration (Bloom, 2014). It is known that neurons rely on microtubule-dependent transport machinery for survival and development. Failure of axonal transport has been proposed as a key contributor in the progression of neurodegenerative disorders such as AD (De Vos et al., 2008; Vicario-Orri et al., 2015). A number of studies suggest that axonal swellings consisting of accumulated abnormal amounts of microtubule-associated proteins, organelles, and vesicles are the important early pathological features in mouse AD models and human AD (Stokin et al., 2005).

Reductions in microtubule-dependent transport may increase Aβ levels and amyloid plaques deposition in AD. The Aβ peptides are proteolytic fragments of the type I transmembrane receptor-like APP protein that plays a role in promoting neurite outgrowth (Sosa et al., 2017). APP is axonally transported, endocytosed and sorted to different cellular compartments where APP is cleaved by several secretases to generate Aβ (Tang, 2009). The kinesin-dependent APP axonal transport is affected by HAP1 in neurons, and lack of HAP1 decreases the transport of APP vesicles from the cell body to neuritis (McGuire et al., 2006). HAP1 and APP have been found to be highly colocalized to interact with each other in mice and human brain (Yang et al., 2012). HAP1 regulates APP trafficking to the non-amyloidogenic pathway and reduces Aβ level in neurons (Yang et al., 2012). HAP1’s close partner AHI1 also participates in APP trafficking and processing to rescue AD pathology (Ting et al., 2019). Recent study suggests that transport of endosomes and vesicles containing APP and Trks is inhibited in AD mouse models (Lazarov et al., 2007). Deficit of APP trafficking affects neurotrophic signaling as APP interacts with the NGF receptors Trks and mediate neuronal survival and differentiation (Zhang et al., 2013). In line with this, HAP1 is important for the stability and sorting of Trks (Rong et al., 2006; Xiang et al., 2014), and HAP1 reduction selectively affects survival and growth of postnatal mice (Xiang et al., 2014). In conclusion, APP-related transport mechanism is a potential therapeutic strategy for AD, in which HAP1 may play an important role.

### The role of HAP1 in other NDs

HAP1 participates in the neuropathology of certain subtypes of SCAs, a group of autosomal dominantly inherited neurodegenerative disorders, that share the characteristic of progressive ataxia resulting from degeneration of cerebellum and its connections. There are over 40 SCAs, including common SCAs (SCA1, SCA2, and SCA3/Machado-Joseph, SCA6, SCA7, and SCA17) and dentatorubral-pallidoluysian atrophy (DRPLA), all of which are caused by an expansion of a CAG trinucleotide repeat in the distinct disease genes (Soong and Morrison, 2018). HAP1 has been reported to associate with TATA binding protein (TBP) and ataxin-3, which are the causative agents of polyQ-expansion-dependent neuropathology of SCA17 and SCA3, respectively (Prigge and Schmidt, 2007; Takeshita et al., 2011). In SCA17 patients, overaccumulation TBP assembles nuclear aggregates, resulting in neuronal loss (van Roon-Mom et al., 2005). HAP1 binds specifically to the conserved C-terminal domain of TBP to prevent nuclear localization of excessive TBP by sequestering a subset of TBP into STBs (Prigge and Schmidt, 2007). Analogously, HAP1 interacts with ataxin-3 intracellularly through its N-terminus Josephin domain and modifies its pathophysiological involvement in SCA3 (Takeshita et al., 2011).

HAP1 also seems to be involved in the neuropathology of SBMA, a neurodegenerative and neuromuscular genetic disease characterized by progressive proximal (bulbar and limb) muscle atrophy, weakness and fasciculations, that is caused by the expansion of a polyQ-encoding CAG tract in the *AR* gene (Takeshita et al., 2006; Sengupta et al., 2022). HAP1 can interact with AR through its ligand-binding domain in a polyQ-length-dependent manner and protect cells from polyQ-expanded AR-induced apoptosis by sequestering polyQ-expanded AR in STBs (Takeshita et al., 2006). HAP1/STB critically involves in pathogenesis of SBMA as an important intrinsic neuroprotectant determining the threshold for cellular vulnerability to apoptosis.

The role of HAP1 in PD and ALS has been less reported. PD, the second most common degenerative disease of the central nervous system, is characterized neuropathologically by loss of dopaminergic neurons and formation of α-synuclein (α-Syn)-containing Lewy bodies in the substantia nigra, manifesting as reduced facilitation of voluntary movements (Spillantini et al., 1997; Damier et al., 1999). ALS, also known as motor neuron disease, is primarily characterized by progressive loss of motor neurons in the brain and the spinal cord, which leads to muscle weakness and eventual paralysis (Hardiman et al., 2017). Accumulation of misfolded proteins, such as α-Syn in PD, TAR DNA-binding protein 43 (TDP-43) in ALS, is also the main event triggering pathological abnormalities responsible for PD and ALS (Soto and Pritzkow, 2018). It would be interesting to investigate whether HAP1 also affects the intracellular trafficking of these proteins to participate in the neuropathology of PD and ALS.

In addition to central nervous system (CNS), HAP1 is also present in the enteric nervous system (ENS) (Tarif and Islam, 2021; Tarif et al., 2022) that is embedded in the wall of the gastrointestinal tract and contains polarized neural circuits responsible for controlling a wide variety of gastrointestinal functions (Furness et al., 2014). The ENS, sharing many features with the brain, can act as a potential portal for pathogenesis of NDs and is also a neurodegenerative target (Chalazonitis and Rao, 2018). HAP1 is abundantly expressed in excitatory and inhibitory motor neurons of myenteric plexuses, and secretomotor and vasodilator neurons of submucosal plexuses (Tarif and Islam, 2021; Tarif et al., 2022). The fact suggests that HAP1-associated intracellular trafficking plays an important role in these neurons of ENS. It is intriguing to consider that the dysfunction of HAP1 in ENS could be a potential risk factor for certain NDs.

## Conclusion

The interactions of HAP1 with cytoskeletal proteins allow HAP1 to participate in the intracellular trafficking of various proteins, stability and recycling of membrane receptors, nuclear translocation of transcription factors and nuclear receptors. Thus, acting as a scaffold to link microtubule transporters with different cargos, HAP1 may stabilize protein complexes that are required for protein trafficking to the appropriate cellular sites. It could also function as an adaptor for two or more components of a signaling pathway. HAP1 interacts with a number of proteins that are involved in NDs, such as HTT, TBP, ataxin-3, and AR. Abnormal interactions of mutant proteins with HAP1 affect the intracellular transport of selected molecules, which is an important contributor to the pathogenesis of NDs. For example, HAP1-dependent intracellular trafficking could be affected by the abnormal interaction of HAP1 with mHTT, as HAP1 binds mHTT more tightly than normal HTT in HD. On the other hand, many major human NDs, including AD, HD, PD, ALS, SBMA, and SCA, display axonal pathologies including abnormal accumulations of proteins and organelles. Such pathologies highlight damage to axonal transport, a key contributor in the progression of NDs. Given the function of HAP1 in intracellular transport that is vitally important for neuronal function and survival, dysfunction of HAP1 may cause axonal transport defect that can account for a number of NDs. Thus, improving or restoring the function of HAP1 could be a potential therapeutic strategy for treating NDs.

## Author contributions

XC and JX wrote the manuscript. EH, CS, and YZ edited the manuscript. All authors contributed to the article and approved the submitted version.

## Funding

Our research work is supported by grants from Natural Science Foundation of Hubei Province (2022CFB216), Key Research Project of Ministry of Science and Technology of China (2022ZD021160) and National Natural Science Foundation of China (82071272).

## Conflict of interest

The authors declare that the research was conducted in the absence of any commercial or financial relationships that could be construed as a potential conflict of interest.

## Publisher’s note

All claims expressed in this article are solely those of the authors and do not necessarily represent those of their affiliated organizations, or those of the publisher, the editors and the reviewers. Any product that may be evaluated in this article, or claim that may be made by its manufacturer, is not guaranteed or endorsed by the publisher.
